# How do patient information documents present dialysis and conservative kidney management? A document analysis

**DOI:** 10.1093/ckj/sfaf136

**Published:** 2025-05-12

**Authors:** Ryann Sowden, Chloe Shaw, James Robb, Anna Winterbottom, Katherine Bristowe, Hilary L Bekker, James Tulsky, Fliss E M Murtagh, Rebecca Barnes, Fergus J Caskey, Lucy E Selman

**Affiliations:** Palliative and End of Life Care Research Group, Bristol Medical School, University of Bristol, Bristol, UK; Palliative and End of Life Care Research Group, Bristol Medical School, University of Bristol, Bristol, UK; Palliative and End of Life Care Research Group, Bristol Medical School, University of Bristol, Bristol, UK; Leeds Institute of Health Sciences, University of Leeds, Leeds, UK; Cicely Saunders Institute, King's College London, London, UK; Leeds Institute of Health Sciences, University of Leeds, Leeds, UK; Department of Supportive Oncology, Dana-Farber Cancer Institute, Boston, MA, USA; Center for Palliative Care, Harvard Medical School, Boston, MA, USA; Wolfson Palliative Care Research Centre, Hull York Medical School, Hull, UK; Nuffield Department of Primary Care Health Sciences, University of Oxford, Oxford, UK; Bristol Medical School, University of Bristol, Bristol, UK; North Bristol NHS Trust, Bristol, UK; Palliative and End of Life Care Research Group, Bristol Medical School, University of Bristol, Bristol, UK

**Keywords:** conservative kidney management, dialysis, document analysis, kidney replacement therapy, patient education

## Abstract

**Background:**

Most older people with advanced kidney disease face a decision between conservative kidney management (CKM) or dialysis and must weigh their potential benefits, risks and impacts on quality and length of life. Patient information documents are designed to supplement patients’ understanding of their kidney disease and explain treatment options to support decision-making. We aimed to explore how patient information documents frame the treatment options of CKM and dialysis and consider implications for patients’ treatment choice.

**Methods:**

We conducted a qualitative document analysis of patient information documents collected from four UK renal outpatient departments with variation in rates of CKM for people ≥75 years of age. Data were analysed using critical discourse analysis.

**Results:**

Three global themes were identified: 1) Treatment options are not presented equally: Dialysis was constructed as the assumed patient choice. CKM was often omitted as an option; when included, it was always mentioned last and was typically constructed negatively. 2) Deciding is challenging: Treatment decision-making, particularly choosing CKM, was portrayed as a challenge requiring emotional support, with clinicians the ultimate decision-maker. 3) Dialysis is living, CKM is dying: Patient information documents presented patients as living with one treatment option choice (dialysis) and dying with another (CKM). Advance care planning, palliative care and information about dying were presented only in the context of CKM, implying these were irrelevant topics for people choosing dialysis.

**Conclusions:**

Patient information documents presented unbalanced explanations of dialysis and CKM. Dialysis was framed as ‘treatment’ and possible complications were minimized. CKM was framed as ‘non-treatment’ and linked to advance care planning, palliative care and death. Inaccurate framing of both CKM and dialysis may mean patients exclude treatment options that may be more concordant with their goals, values and preferences.

## Graphical Abstract

**Graphical Abstract:**
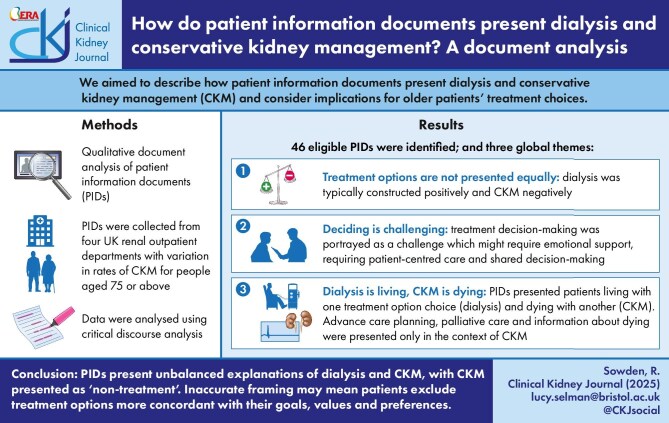

KEY LEARNING POINTS **What was known:**

- Across UK renal units, there is considerable unwarranted variation in rates of CKM and dialysis among older people with kidney failure, suggesting that the guidance that people with kidney disease receive about treatment options varies.
- People with kidney failure receive patient information documents to supplement guidance provided in consultation with their clinicians.
- Previous studies have shown that patient information documents may not provide balanced or clear treatment information; however, depictions of CKM and dialysis in patient information have not been explored in detail.

**This study adds:**

- This study reveals how patient information documents present information relating to CKM and dialysis in unbalanced and unrealistic ways, providing recommendations for how those responsible for the production of patient information documents can rebalance information.
- Patient information documents fail to provide information regarding death, dying and end-of-life relating to dialysis and instead link death, dying and end-of-life care to CKM.
- This study shows how patient information documents, despite referencing ideals of patient-centred decision-making, may subtly undermine this goal by implicitly presenting dialysis as the first choice and CKM as second-best, without fully exploring the potential benefits and drawbacks of each decision relative to individual lifestyles and preferences.

**Potential impact:**

- Our findings show that patients are exposed to unrealistic and unbalanced information about treatment options, which may lead to patients selecting treatment options that are not concordant with their goals, values and preferences.
- Specifically, findings suggest that, based on information leaflets, patients who choose dialysis may have the misconception that it is risk-free, and always life-extending, and that information regarding death, dying and end-of-life care is not relevant to them.
- Study recommendations can help inform the creation of patient information documents that are more balanced and realistic regarding treatment options, supporting patients in the choice of treatment that best suits their individual values, priorities and preferences.

## INTRODUCTION

Only 1% of patients in Europe ≥75 years of age who receive dialysis progress to transplant [1], meaning most older people approaching kidney failure face the decision between preparing for dialysis or conservative kidney management (CKM). CKM aims to delay disease progression and treat symptoms without replacing kidney function [2]. For people ≥80 years of age, or ≥65 years with comorbidities, dialysis provides uncertain or modest survival benefits [3, 4] and has the greatest symptom burden [4] and impact on quality of life [4–6], negatively impacting daily activities and functional dependency [5–7].

Rates of CKM vary significantly between UK renal units; in 2012, the proportion of patients ≥75 years of age receiving CKM ranged from 5% to 95% between units [8]. This variability suggests treatment decision-making is inconsistently guided by the evidence base and that decision-making support is not consistently person-centred [9, 10], contrary to recommendations [11, 12]. How clinical staff communicate with patients and caregivers regarding treatment options is highly variable across renal units [13–16]. At units with a more established CKM pathway, patients were more aware of CKM and less often believed that dialysis guaranteed longevity [17].

Patient information documents (PIDs), available in renal care settings and online, are produced by clinicians, charities, dialysis companies and other stakeholders such as pharmaceutical companies as a supplement to clinical guidance [8, 18, 19]. PIDs inform treatment decision-making and help patients and families prepare for treatment [15, 20, 21]. PIDs may be particularly valuable for patients with cognitive impairment, common in kidney failure [22], providing the opportunity to clearly present information in an accessible way. However, PIDs are often incomplete and difficult to understand [20].

Decision aids are one type of PID, using decision science to support patients’ understanding of medical problems and treatments [23]. Decision aids have been specifically developed to support patients’ renal decision-making [19, 24, 25], but there are implementation barriers [26, 27] and they are not widely used [28, 29] or are provided only when treatment decision-making is imminent. However, patients are exposed to a range of PIDs from their initial referral to nephrology (e.g. in outpatient waiting rooms), long before a treatment decision might need to be made, hence understanding their framing of treatments and implications for patients’ treatment decision-making is crucial. ‘Framing’ can be understood as the meaning conveyed about a topic from the way in which it is communicated [30]. Framing stresses certain aspects of reality and pushes others into the background through the processes of selection and salience, with consequences for how a subject is understood or interpreted.

We aimed to explore how PIDs frame the treatment options of CKM and dialysis and any variation between renal units in the information their PIDs provide and consider how this might influence patients’ treatment decisions.

## MATERIALS AND METHODS

### Study design

We conducted a qualitative document analysis of PIDs collected as part of the Optimising Staff-Patient Communication in Advanced Renal Disease (OSCAR) study a mixed methods intervention development study to enhance how renal clinicians communicate with older patients (age ≥65 years) with advanced kidney disease [estimated glomerular filtration rate (eGFR) ≤20 ml/min/1.73 m^2^] regarding their treatment options [31]. An eGFR ≤20 ml/min/1.73 m^2^ was used in OSCAR as this is when clinicians often first raise discussion of a treatment decision [8].

### Data collection

Data collection occurred in four hospitals in England and Wales (June 2021–January 2023), purposively sampled to ensure diversity in kidney failure treatment rates for older people and patient ethnicity (Table 1). The Conservative Kidney Management Assessment of Practice Patterns Study (CKMAPPS) data regarding treatment rates at the sites [8] was updated prior to the study start and are presented in Table 1, as are data collected from sites on the ethnicity of their patient population. Physical and digital PIDs were collected as part of an ethnographic study involving several visits to each site (68 h of observation in total across the sites). The researchers collected physical PIDs from renal outpatient waiting areas and consultation rooms frequented by patients with advanced kidney disease and physical and digital PIDS from clinicians at each site who support treatment decision-making (consultants, registrars and specialist nurses), who were asked which PIDS they used with patients.

**Table 1:** Characteristics of the four sites.

Characteristics	Site 1	Site 2	Site 3	Site 4
Hospital type	Regional teaching hospital	Regional teaching hospital	Local general hospital	Regional teaching hospital
Hospital size (inpatient beds)	≥1000	≥2000	≥500	≥1500
Presence of a low-clearance clinic	Not at main hospital, but at some satellite sites	Yes	Yes	Yes
Black, Asian and minority ethnic patient ethnicity % (UK Renal Registry, 2022) [63]	12.1: South Asian 3.0 Black 6.7 Other 2.4	10.2: South Asian 6.2 Black 0.6 Other 3.4	3: South Asian 3.0 Black 0.0 Other 0.0	57.7: South Asian 21.0 Black 26.2 Other 10.5
Patients ≥75 years receiving CKM % (calculated from CKMAPPS data, 2012) [8]	16	28	1–9	45
Patients with CKD stage 5 age ≥75 years in 2016 *n*	350	82^a^	120^a^	318
CKD stage 5 patients age ≥75 years receiving CKM in 2016 *n* (%)	182 (52)	36^a^ (44)	15–20^a^ (13–17)	115 (36)

^a^Estimated by site leads from hospital records where exact data not recorded.

### Inclusion and exclusion criteria

PIDs were included if they discussed CKM, dialysis (including haemodialysis (HD) and peritoneal dialysis (PD)) or both. PIDs were categorized as direct treatment option information resources (category A) when focussed on treatment options individually or in comparison, or indirect information resources (category B) when focussed on broader contextual information related to treatment options, including lifestyle information and treatment logistics.

### Analysis

The analysis examined how PIDs describe and explain living and dying with kidney failure in relation to the treatment options of CKM and dialysis, considering how this might affect patients’ decision-making. We considered how treatment options were contextualized, identifying themes, ‘frames’ and discourse [32]. Text and images were coded with a focus on identifying manifest themes (types/aims of the PIDs; framing of the content, including images), latent themes (implicit content) and differences by treatment type and renal unit.

The research team included social scientists specializing in communication, decision science, kidney disease and palliative care and clinical academics in nephrology and palliative care. Our analytic approach was consistent with critical discourse analysis [33], identifying and examining ideologies embedded in discourse [34, 35]. Our analytical process involved reading, coding and interpreting each of the PIDs, identifying recurring narrative patterns and themes and considering differences between renal units. R.S., C.S., L.E.S. and J.R. independently created draft coding frameworks by inductively coding three category A and three category B PIDs. After discussion, comparison and refinement, a hierarchical coding framework was agreed upon and applied to all PIDs, using NVivo 12 (Lumivero, Denver, CO, USA) for data management. R.S. or J.R. applied the coding framework to each PID and then met to review and check all coding. R.S. developed an analytic narrative, refined with the research team, drawing on Dixon-Woods’ identification of two discourses in PIDs: patient education discourses, which originate from the biomedical perspective, assume patient incompetence and use a mechanistic model of communication; and patient empowerment discourses, which value patient knowledge and focus on shared decision-making [36]. The team discussed how the distinct narratives identified were likely to influence patients’ understanding of and selection of treatment options.

Data extracts are tagged according to category (A = direct treatment option information resource, B = indirect information resource), site of collection (site 1–4) and document number (e.g. A-1-10 is a direct treatment option information resource collected from site 1 and the 10th PID collected from that site).

### Ethical considerations

Ethical approval for the OSCAR study was granted by the Health Research Authority Bromley Research Ethics Committee (21/LO/0280).

## RESULTS

**Figure 1:**
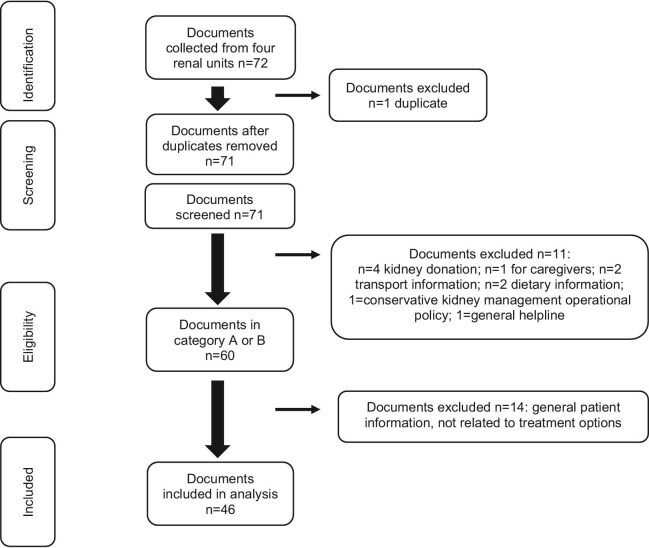
Flow diagram—identification of patient information documents.

A total of 72 PIDs were identified and 46 PIDs were included after deduplication and screening (Fig. 1): 29 direct treatment option information resources and 17 indirect information resources (Table 2). PIDs included leaflets (*n* =  38), posters or flyers (*n* = 4), magazines (*n* = 2) an app (*n* = 1) and a decision aid (*n* = 1). Across the sites, PIDs included a range of in-house (*n* = 14) and nationally produced (*n* = 32) PIDs.

**Table 2:** Characteristics of PIDs (*n* = 46).

Characteristics	ID code	Treatments mentioned *n*	Treatments mentioned	How CKM is referred to	Locally or nationally produced
**A: Treatment option information resources**					
Site 1 documents (*n* = 9)	B10	2	HD TR	N/A	National
	B11	2	PD HD	N/A	National
	B18	2	D CKM	Not having dialysis	Local
	B19	2	TR D	N/A	Local
	B20	4	HD PD TR CKM	Conservative care	National
	B3	2	HD PD	N/A	National
	B7	2	PD HD	N/A	National
	B8	4	HD TR PD CKM	Conservative care	National
	B9	3	PD TR HD	N/A	National
Site 2 documents (*n* = 7)	C1	2	D CKM	Choosing not to have dialysis	National
	C10	2	D CKM	Conservative management	Local
	C2	3	TR D CKM	No dialysis	National
	C3	2	D TR	N/A	National
	C5	2	HD CKM	Conservative management	Local
	C8	1	PD	N/A	Local
	C9	2	CKM D	Conservative management/supportive care	Local
Site 3 documents (*n* = 8)	G10	4	TR HD PD CKM	No dialysis	National
	G11	1	HD	N/A	National
	G17	2	D TR	N/A	National
	G20	1	D	N/A	National
	G21	1	HD	N/A	National
	G6	1	D	N/A	National
	G7	3	D TR CKM	Conservative medical management	National
	G9	2	D TR	N/A	National
Site 4 documents (n = 5)	L12	2	D CKM	Not having dialysis, conservative, supportive	National
	L14	4	PD HD TR CKM	Maximum conservative management	Local
	L2	3	TR D CKM	Conservative management	National
	L4	4	PD HD TR CKM	Choosing not to have dialysis or a transplant	National
	L5	3	D PD TR	N/A	Local
**B: Indirect information resources**					
Site 1 documents (*n* = 7)	B1	1	HD	N/A	Local
	B12	1	D	N/A	National
	B14	1	HD	N/A	Local
	B15	3	HD PD TR	N/A	National
	B16	3	HD PD TR	N/A	National
	B17	2	D TR	N/A	Local
	B6	1	D	N/A	Local
Site 2 documents (*n* = 2)	C4	1	D	N/A	National
	C7	1	HD	N/A	National
Site 3 documents (*n* = 5)	G12	3	HD PD TR	N/A	National
	G22	1	D	N/A	National
	G4	2	D TR	N/A	Local
	G5	1	D	N/A	Local
	G8	1	D	N/A	National
Site 4 documents (*n* = 3)	L6	1	D	N/A	National
	L8	1	PD	N/A	National
	L9	1	HD	N/A	National

CKM: conservative kidney management; D: dialysis; TR: transplant; HD: haemodialysis; PD: peritoneal dialysis; N/A: Not applicable as CKM not mentioned.

Analysis identified 3 global themes, 9 themes and 41 subthemes (Table 3). These are discussed with reference to linguistic aspects (use of language, vocabulary, grammar), layout and patient education and empowerment discourses [36]. Discussion focusses on category A PIDs, which formed most of the dataset, with category B PIDs used to supplement findings.

**Table 3:** Themes, subthemes and exemplars.

Global theme	Themes	Subthemes	Exemplar	Code
1. Treatment options are not presented equally	Treatment is dialysis	Dialysis is life-saving	‘Kidney failure, if left untreated, is fatal but modern medicine has provided us with a life-saving treatment—DIALYSIS.’ A-3-11 (Site 3)	Q1
		Dialysis implicit choice	**‘Will I need dialysis for the rest of my life?**	Q2
			As long as you have no renal function you will need dialysis’. A-1-10 (Site 1)	
	CKM is non-treatment	Stopping kidney replacement therapy equated with CKM	‘There are separate information leaflets about haemodialysis, kidney transplantation and stopping dialysis available on the Renal Association and Kidney Care UK websites’. A-1-09 (Site 1)	Q3
		CKM equated with death	‘Some people may decide not to have dialysis and this will result in death from kidney failure’. A-1-10 (Site 1)	Q4
		Negative descriptions of CKM	‘Some patients may have been advised that dialysis would not be in their best interest due to their complex medical problems or they may feel they do not want to have dialysis treatment’. A-2-5 (Site 2)	Q5
		Attempts to validate choice	‘Some people with kidney failure may have lots of other serious health issues. For these people, having dialysis or a transplant may not make them feel better, or improve their quality of life’. A-3-07 (Site 3)	Q6
		Ethics of CKM	‘Stopping dialysis is not suicide nor is it euthanasia or assisted dying. It is withdrawing from renal replacement treatment.’ A-1-18 (Site 1)	Q7
		Family don't like choice	**‘It is very difficult to know how to talk to my family and friends. Can you help?**’	Q8
			Yes. Many people find it difficult to talk about such matters to those who are close to them’. A-1-18 (Site 1)	
2. Deciding is challenging	Emotional support	Empathic statements e.g. hard to choose, hard coming to terms with kidney failure	‘Being diagnosed with kidney failure can be a very worrying time for patients and their families. It marks the beginning of a life-long “career” as a kidney patient and starts a journey of decision-making about treatment choices’. A-3-10 (Site 3)	Q9
		Choosing CKM requires support	**‘Who can I talk to if I decide not to start dialysis treatment?**’	Q10
			‘It is important for you and your family to talk these matters through with members of the renal team. It is best if this is a decision that has been made together’. A-1-18 (Site 1)	
		Difficult to discuss	‘**It is very difficult to know how to talk to my family and friends. Can you help?’**	Q11
			‘Yes. Many people find it difficult to talk about such matters to those who are close to them. They are often concerned that they will upset their relatives or worried how they will react. With anything that is difficult, it is always best to talk openly and honestly’. A-1-18 (Site 1)	
		Availability of emotional support	**‘Who should I talk to if I am considering opting for MCM (maximum conservative management)?’**	Q12
			... There are a number of people who can help you: - The doctors and nurses looking after you looking after you [sic] will give you every opportunity to discuss the options in clinic and ask any questions you may have. - You can also discuss your feelings and concerns with our psychologist’. A-4-14 (Site 4)	
	Clinical values and principles	Empowering/paternalistic	‘The exact timing varies from person to person and may depend on medical factors and the underlying kidney problem. Your nephrologist will ultimately decide when you need to start dialysis’. A-2-05 (Site 2)	Q13
		Right to decide	**‘Can I choose not to have dialysis treatment?’**	Q14
			‘Yes. You have the right to decide not to start treatment if you feel the burden of dialysis would outweigh the benefits to you and lessen your quality of life’. A-1-18 (Site 1)	
		Informed decision-making	**Making an informed choice**	Q15
			‘If your kidneys fail, there are a number of treatment options to choose from. However certain medical and social factors will need to be discussed with you and your family/carer to help you decide what is best for you’.	
			‘Your Specialist Nurse can help explore the most appropriate choice of dialysis with you. She can discuss your individual issues and worries with you and help you to plan ahead’. A-2-05 (Site 2, underlining added for emphasis)	
		Need for accuracy and honesty	‘In this leaflet, we aim to give you an honest and accurate account of what might happen if you do not have dialysis. We will describe the symptoms of advanced kidney disease and how they can be managed without dialysis. We also aim to answer some of the questions that you might find difficult to ask’. A-1-18 (Site 1)	Q16
		Patient participation	**Patient view**	Q17
			You can sign up (via your Clinical Nurse Specialist) to access your blood test results, this puts you in more control of your condition/treatment as you become familiar regarding what is normal for you’. A-3-10 (Site 3)	
		Shared decision-making	‘The team at the renal unit will work together with you’.	Q18
			‘They will share their knowledge to help you make the best decision to fit with your lifestyle’. A-3-07 (Site 3)	
		Patient goals and values	**‘Kidney failure: What choices do I have?’**	Q19
			... ‘One of the best things that you can do before making any decision about your treatment is to find out as much as you can about what choices are available to you locally, what choices will suit the way you live your life and whether you want to travel for holidays’. A-3-10	
	Making a choice	The best choice	‘The best way to make a choice about which dialysis treatment will suit you is to talk to the staff and to other patients about their decisions and what they like and don't like about their treatment choice. It is usually possible to change your type of dialysis if you want to after some planning’. A-2-02 (Site 2)	Q20
		Decision permanency—time and circumstance relevant	‘As established kidney disease is a life-long condition, people may change their treatment choice as their disease and life changes. It is likely people will make different decisions at different points in their life’. A-1-20 (Site 1)	Q21
		Making choice with others	‘Your kidney doctors and staff should help you choose the treatment that is right for you’. A-3-10 (Site 3)	Q22
		Trials	‘Sometimes it may not be clear if the benefits outweigh the burden of the dialysis. In this case you could start dialysis for a trial period. You can make you decision after that’. A-3-07 (Site 3)	Q23
	Certainty and risk	Risk	‘CKD can slowly get worse over time, although for the majority of people it remains stable and only a very small number of people will need renal replacement therapy such as dialysis. It is unusual for kidney function to improve dramatically once your kidneys have been damaged but it does depend on the cause of the problem’. A-3-6 (Site 3)	Q24
		Complications	‘Dialysis itself has complications. One of the most common complications of infection as haemodialysis needs access to the blood stream. Patients on haemodialysis have a higher risk of heart attacks and strokes. It can be difficult to reduce the fluid intake as much as is required, leading to fluid on the legs and lungs’.	Q25
			‘Most patients experience some of these complications…but that does not mean you're bound to get all of them!’ A-1-10 (Site 1)	
		Older age	‘[CKM] might not lengthen life for people who are older, have other long-term illnesses and have difficulty looking after themselves’. A-1-20 (Site 1)	Q26
		Frailty	‘There is increasing evidence to show that if a person is older and has significant medical issues dialysis does not offer any (or little) benefit in either quality of life improvement or survival times. This will be discussed with you by your Nurse Specialist and Nephrologist’. A-2-05 (Site 2)	Q27
		Comorbidity	No dialysis	Q28
			‘Some patients choose a path called conservative treatment rather than treatment with dialysis. This may suit people with other medical conditions who feel dialysis is not for them’. A-3-10 (Site 3)	
		Uncertainty	Approaching failure in kidney function	Q29
			‘…Patient risk of progression to kidney failure requiring dialysis or transplant’. A-1-21 (Site 1)	
	Quality of information	Accuracy of clinical	‘**Is dialysis treatment painful?’**	Q30
		information	‘Once you get used to the needles being placed, dialysis is not painful but can be uncomfortable’. A-1-10 (Site 1)	
		Framing and goals of treatment options	‘In such situations, it is important for all concerned to have a clear understanding of the likely advantages and disadvantages of undertaking dialysis treatment. This should take account of your particular problems circumstances and concerns’. A-1-18 (Site 1)	Q31
3. Dialysis is living, CKM	Living with the	Changes/staying the same	**Living with kidney failure**	Q32
is dying	treatment option	and presentation of living	‘Becoming a dialysis patients is a turning point in your life. Modern medicine makes it possible for you to live and continue with many day-to-day activities’.	
			‘As patients, we know that the path will not always be easy. Dialysis is now part of your life and that of your family. Do not let it dominate—the aim is to enable you to enjoy a good quality of life. Above all, at [organisation]we aim to show that living with kidney failure can be life well worth living—why not join us?’ A-1-10 (Site 1)	
		Symptoms of kidney failure and their management	‘The symptoms associated with Chronic Kidney Disease may include: nausea, poor appetite, breathlessness, swollen ankles, restless legs, itching, cramps, severe tiredness, low mood, occasionally pain, or rarely seizures’.	Q33
			‘If your symptoms become more troublesome we will liaise with your GP to discuss medications that may be beneficial. If appropriate we may also involve the local palliative care team who are specialists in managing such symptoms. We can also contact organisations who can help you with financial matters or offer psychological support’. A-2-10 (Site 2)	
	Dying with the treatment option	Timescale		Q35
			**‘How long can you live on dialysis?’**	
			‘We do not yet know how patients on dialysis will live. We think that some dialysis patients may live as long as people without kidney failure’. A-3-11 (Site 3)	
		Description of dying	‘What happens at the end?’	Q36
			‘As you get towards the end of your life, the symptoms that you already have may gradually get worse. In particular, you may find you spend more time in bed and have less energy. Those looking after you will continue to address any symptoms you have and try to relieve them. Some people become more drowsy and their thoughts less clear as they become more sleepy’. A-1-18 (Site 1)	
		Planning	**Choosing not to have dialysis or transplant**	Q37
			**Conservative and end-of-life-care**	
			‘This is not the same as end of life care. This term is used to refer to the time period and care needed when someone's life is coming to an end. It may be someone who has not decided to start dialysis at all. For others it may be that they have had dialysis for many years but become frail or disabled and have decided their time has come to stop treatment and die with dignity’. A-4-14 (Site 4)	
		Introduction to palliative care	‘When these changes to people's health happen, kidney professionals offer treatments to relieve people's symptoms and give some comfort. These types of options are sometimes known as palliative care’. A-1-20 (Site 1)	Q38
		Advance care planning (ACP)	**What support and help will there be as my kidney function gets much worse?**	Q39
			**Your doctor or nurse may suggest talking to specialists in end of life care.**	
			‘These doctors and nurses are known as the palliative care team and they will talk to you about how you would like to be looked after as your kidney function declines. These discussions are known as Advance Care Planning. They may involve your GP, your family and staff from your local hospice’. A-2-01 (Site 2)	

### Treatment options are not presented equally

The treatment option of dialysis was conceptualized as an explicitly ‘life-saving’, death-preventing and life-prolonging treatment that patients could receive indefinitely [assuming no complications (Q1)]. Discussions of death or end-of-life care were largely absent from descriptions of dialysis.

Dialysis was constructed as the assumed patient choice. Some PIDs were labelled as published by the ‘pre-dialysis service’ (e.g. A-2-05/09) rather than the renal service, even when PIDs described or focused on CKM. Low kidney function was equated with a ‘need’ for dialysis specifically (Q2). When listing treatment options, CKM was often completely absent. When CKM was included as a treatment option, it was always the last treatment mentioned (regardless of whether dialysis was described generally or specifically as HD or PD).

Where CKM was included, it was also poorly and inconsistently defined. It was inconsistently named across PIDs and sometimes within the same PID, being referred to variously as ‘conservative treatment’, ‘supportive care’, ‘choosing not to have dialysis’ and ‘maximum conservative management’ (Table 2). CKM was often presented as the antithesis of dialysis and commonly described using negation (e.g. ‘non-dialysis’, ‘non-treatment’). CKM was framed as opting out of the default treatment (dialysis) or erroneously equated with stopping dialysis (Q3). Even descriptions of its possible advantages framed CKM as a direct challenge to the default position of dialysis: ‘Why would I choose not to have dialysis?’ (A-2-01).

In addition, CKM was routinely linked to death and dying: explicitly, in descriptions of death from kidney disease (Q4); implicitly, by presenting CKM in proximity to discussion of palliative care and advance care planning (ACP). CKM was described as appropriate for patients who had been ‘advised that dialysis would not be in their best interests’, akin to drawing the short straw (Q5). Occasionally, CKM was more positively presented for people with additional comorbidities (Q6), and there were attempts to legitimize and validate CKM, providing reassurance that CKM is an ‘ethical choice’ distinct from assisted suicide (Q7).

CKM was also portrayed as not preferred or understood by patients’ family members. One PID informed patients that a ‘family conference’(A-4-14) could be organized if a patient had chosen CKM and their family were not supportive (Q8).

Images used to depict dialysis and CKM supported these conceptualizations. Dialysis was typically illustrated with colourful photos of smiling, healthy, younger people enjoying life in non-medical settings or, sometimes, on dialysis machines. CKM was typically illustrated by older people, looking sad or tired. Sometimes the vagueness of information regarding CKM was mirrored in the use of unspecific clip art. Occasionally the visual presentation of CKM was comparable to the positive depictions of dialysis, e.g. A-3-07 depicted CKM in a way that implied it allowed free time.

### Deciding is challenging

PIDs presented treatment decision-making as challenging, frequently including empathic statements regarding the difficulty of choice (Q9). CKM was often singled out as requiring additional discussion with clinicians, family members or both (Q10). PIDs characterized CKM as inherently difficult to consider or discuss (Q11), potentially requiring psychological support (Q12). A choice of CKM was presented as something to be discussed and tested, with clinicians portrayed as paternalistic, ultimate decision-makers (Q13). Where PIDs made explicit reference to a patient's right to choose CKM, it was framed as relevant when dialysis was found too burdensome (Q14).

Another PID highlighted the importance of informed decision-making. Roles were constructed: clinicians as educators, patients as information receivers and deciders (Q18). The tone was suggestive of patient passivity (Q15). In line with these roles, PIDs often referred to the need for clinicians to give accurate and honest information (Q16), while patients were presented with optional roles in looking after their kidneys (Q17). Some PIDs invited patient participation directly e.g. encouraging patients to make notes within PIDs.

Discussion of patients’ personal circumstances generally focussed on problems/concerns (Q31). Preferences, goals and values were only raised implicitly, in relation to patients’ lifestyle. Q19 shows how consideration of treatment options (rather than a default assumption of dialysis) can help present the decision in a more neutral, patient-centred way.

Ideal patient decision-making was presented as ‘involving weighing advantages and disadvantages and discussion with other people’ (Q20): always clinicians (Q22), sometimes loved ones, infrequently other patients. One PID insisted on the need for external help: ‘it is impossible to manage on your own’ (A-2-02).

Overall, information regarding the status of the decision and the nature of the treatments was inconsistent and sometimes inaccurate. While some PIDs indicated that decisions were not static, but depended on time and circumstance (Q21), others implied that the choice of treatment was permanent until death. Trials of dialysis were infrequently described and always in the CKM section of a PID (Q23). Information on treatment complications (e.g. infection, success rate) was often absent or minimized (Q25). PIDs usually omitted information regarding prognosis and the associated risks and uncertainties of each treatment option. Where included, uncertainty and risk were typically framed in terms of potential complications related to age (Q26), frailty (Q27), comorbidity (Q28) and ‘worst case scenarios’, alongside reassurance (Q24). Overall, dialysis was portrayed as painless (Q30), with minimal side effects and negative aspects limited to travel and boredom.

### Dialysis is living, CKM is dying

The theme ‘Living with the treatment option’ (typically dialysis) described how a person's life could be expected to change or stay the same. ‘Dying with the treatment option’ (typically CKM) described timescale and life expectancy.

PIDs’ presentation of living with treatment focused on the ability to maintain a usual lifestyle. For PIDs describing dialysis, continuation of work, holidays and hobbies were presented as common, possible and easy. Few PIDs acknowledged the potential challenges of maintaining life as usual; those that did typically minimized the challenges by outlining a need for acceptance (Q32).

The symptoms and management of kidney failure were commonly presented in a distinct section, but occasionally under CKM, implying symptoms from kidney failure were experienced only if CKM was chosen and not alongside dialysis (Q33). PIDs discussed life expectancy more commonly in relation to CKM than dialysis, with inconsistent messaging (Q27, Q35). CKM was often associated with a shorter lifespan and dialysis was presented as a treatment that could continue ‘forever’, providing a life expectancy comparable to someone without kidney failure. Conversely, some PIDs noted that while there was variation, life expectancy could be similar for CKM and dialysis. This information was less common in PIDs from site 3. One PID described survival on dialysis as influenced by comorbidity and lifestyle, as well as ‘luck!’ (A-1-11).

Descriptions of dying were typically absent, or tended to appear in relation to CKM. For patients who chose CKM, death was presented as a certainty and was generally described as ‘peaceful’ (Q36). Typically, end-of-life care was discussed in relation to planning lifestyle adjustments or withdrawal from dialysis (Q37). Where palliative care was introduced, it was often conflated with CKM (Q38). Several PIDs offered general suggestions regarding planning for death and, more specifically, for advance care plans (Q39), but this was only seen in CKM-focused PIDs or under the CKM section of generalized PIDs.

### Similarities and differences

Almost all PIDs focused on providing information about treatment options (rather than explaining low kidney function and why many patients might start to consider treatment options). Discussion of patients’ goals and values was typically absent.

There were some differences by site. Site 1 PIDs were more likely to focus on one specific treatment option without comparison (although site 1 was also the only site to feature a decision aid comparing treatments). Site 3 had a greater number of PIDs produced by a dialysis company and more patient voice resources such as kidney charity magazines or peer support information. Site 4 PIDs generally had an increased focus on emotional well-being and psychosocial support and provided relatively more balanced information for CKM and renal replacement therapy.

## DISCUSSION

This analysis of PIDs from four UK renal units identified a clear bias towards dialysis over CKM. Dialysis was constructed as the default, preferential treatment option. CKM was often omitted as an option; when included, it was inconsistently named, always mentioned last and typically framed as opting out of default treatment and constructed negatively. By omitting information on ACP, palliative care and the end of life in relation to dialysis, PIDs implied these topics are not relevant to people who choose dialysis. Simultaneously, PIDs implied that patients who choose CKM will die from kidney failure, which is why loved ones often object to the treatment and why clinicians (including psychologists) may question this choice.

Biased presentation of treatment options may lead some patients to discount CKM as a treatment option, even when closely aligned with their goals and values, leading to unwanted, futile care [37]. Conversely, patients who choose dialysis on the basis of information from PIDs may have unrealistic expectations of life expectancy and quality of life. In the event of death from kidney disease, this lack of information may lead to poor bereavement outcomes for significant others [38].

PIDs play one part in informing patients about treatments; how clinicians discuss treatment options and communicate about the decision is another crucial element. Our findings support evidence that CKM is commonly framed as the absence of treatment, without its own standardized name, both in leaflets and in how clinicians communicate about treatment options [19, 31]. CKM was referred to variously as ‘conservative treatment’, ‘supportive care’, ‘choosing not to have dialysis’ and ‘maximum conservative management’. This leads to confusion about what CKM is [39], undermining its status as a viable choice [2, 31]. In our analysis of video-recorded real conversations of clinicians discussing treatment options with older patients, published separately [31], we similarly found a bias towards presenting dialysis as the default option for kidney failure and CKM as a subordinate option. Findings from this analysis suggest that PIDs reinforce this message.

Similarities in PIDs between sites point to commonalities in the culture of renal care. However, we also found differences—most notably, site 4 PIDs contained relatively more balanced and holistic information regarding CKM and dialysis, which may help explain relatively high treatment rates among older people at this unit (45% CKM in 2012, 36% in 2016). But given similar rates at sites 1 and 2, at least in 2016 estimates, further research alongside accurate tracking of CKM treatment rates is needed to understand the relative contribution of PIDS (compared with, for example, information from clinicians [31]) to patients' treatment decision-making [8].

Previous studies have also found that patients receiving renal replacement therapy are unequally informed of treatment options [40, 41], leading to dissatisfaction. There is evidence that kidney disease decision aids are rarely tailored to older patients with kidney failure, and similarly lack detail about prognosis and ACP [20, 42]. Ultimately, a lack of accessible, comprehensive and accurate information means a lack of informed choice [13]. In this regard, there is a tension between PIDs’ use of patient empowerment discourses, making explicit reference to the need for honest, accurate and clear information due to the uncertainties associated with each treatment option [43] and the omission of key decision-making information [44]. Dixon-Woods reports that paternalistic obfuscation of clinical uncertainty is typical of clinical information resources [36]. Building on her argument that patient education discourses view and treat patients as incompetent, omitting information can be seen as creating incompetence in the form of misconceptions about the different treatment options.

Overall, we found few explicit discussions in PIDs of prognosis, ACP and end of life. These topics are also often absent from renal consultations [45, 46], despite many patients’ wishes to discuss and receive end-of-life information [47], suggesting the PIDs serve to reinforce biased messages that patients also receive from elsewhere. Kidney patients are often unaware of their prognosis [48] and discussions of palliative care occur too late [49], with evidence of high levels of unmet palliative care needs in advanced kidney disease [50]. Including these crucial topics in PIDs would support treatment decision-making, potentially reducing decisional regret [51] and improving bereavement outcomes [38].

Findings from this study support existing evidence that PIDS in medicine are not always reflective of clinical best practice and evidence or patient need [52–55]. Reasons for the evident biases we identified are likely to be multifaceted and may include clinicians’ discomfort with uncertainty and acknowledging the limits of medical care [56]. PIDs may also be written in anticipation of patients’ negative preconceptions of any modality of dialysis [2], hence aiming to emphasize the ability to live a normal life on dialysis. Alternatively, PIDs may aim to reflect patient priorities such as holidays; however, descriptions of treatments and possible side effects such as pain need to be accurate. A systematic review indicated 63% of patients receiving dialysis experienced pain [57].

Given the requirement to provide integrated care for people with kidney failure which includes supportive care and expert communication [58–60], study findings have important clinical implications. Across the four sites we found a wide variety of PIDs, highlighting inconsistencies in information provision, with only one PID appearing across two sites. Renal units should carefully evaluate the PIDs they provide, considering whether they are balanced, accurate and comprehensive, including covering the implications of treatment choice for end-of-life experiences and care. Ideally, to enhance consistency and reduce variation across renal units, PIDs that have been reviewed and approved nationally should be used. However, a review and redesign of nationally available PIDs is essential, recognizing that even PIDs not explicitly focused on treatment decision-making still influence those decisions. This redesign should ensure PIDs are evidence-based, involve co-production with patients and significant others from diverse ethnic, cultural and religious backgrounds [36, 61], and draw on decision aid methodology to ensure accurate, balanced presentation of treatment options. A standardized term for the treatment pathway needs to be used; ‘conservative kidney management’ is recommended [59]. To align PIDs with their stated values of informed decision-making, end-of-life information should be included. As in previous studies [28, 29], we found decision aids, designed to provide more balanced information regarding treatment options [24, 25], were not readily available or used, with only one identified. Further research is required to identify implementation barriers.

### Limitations

Study strengths include the range of documents analysed from four purposively sampled hospitals selected to ensure diversity in kidney failure treatment rates [8] and patient ethnicity. The inclusion of a range of document types, not just decision aids, allowed for a broader understanding of the information and framing patients are exposed to prior to the ‘need’ to make a treatment decision. Our qualitative findings are not generalizable to all renal units, however through descriptions of settings and samples we have provided details to enable judgement of transferability [62]. Finally, although we included PIDs concerning transplant in our analysis, our focus was on the treatment options of CKM and dialysis as these are the main treatment options for older people with kidney failure.

## CONCLUSION

Once referred to a renal outpatient department, patients are routinely exposed to biased information regarding treatment options. Patient information frames dialysis as ‘treatment’ with minimal side effects and complications and conveys unrealistic expectations of life expectancy and quality of life. CKM is framed as ‘non-treatment’ and explicitly linked to death from kidney failure. Palliative care, ACP and death are discussed only in relation to CKM. Patients may therefore discount CKM, even when it aligns with their individual goals and values. PIDs collected from the renal unit with a relatively high rate of CKM among older patients were more holistic and patient-centred in content, suggesting that PIDs may reflect or reinforce local cultures of care and influence treatment choice.

## Data Availability

The datasets used and analysed during the current study are available from the corresponding author upon reasonable request.
